# A SMYD3/ITGB6/TGFβ1 Positive Feedback Loop Promotes the Invasion and Adhesion of Ovarian Cancer Spheroids

**DOI:** 10.3389/fonc.2021.690618

**Published:** 2021-09-21

**Authors:** Yahui Jiang, Tianyu Zhou, Yiwen Shi, Weiwei Feng, Tianjiao Lyu

**Affiliations:** Department of Gynecology and Obstetrics, Ruijin Hospital, School of Medicine, Shanghai Jiaotong University, Shanghai, China

**Keywords:** ovarian cancer spheroids, metastasis, SMYD3, ITGB6, TGFβ1

## Abstract

**Background:**

Implantation metastasis is the main means of dissemination in ovarian cancer. Our previous studies showed that SET and MYND domain-containing protein 3 (SMYD3) expression was higher in ovarian cancer spheroids than in monolayers. SMYD3 enhancement of spheroid invasion and adhesion is mediated by the downstream effectors ITGB6 and ITGAM. However, the potential mechanisms underlying the SMYD3/integrin-mediated invasion and adhesion of spheroids still need to be explored.

**Methods:**

Western blotting was used to examine the expression of SMYD3, ITGB6 and downstream molecules under different treatments. Immunofluorescence was used to detect the expression of F-actin, E-cadherin and N-cadherin. Anti-ITGB6 antibody-based inhibition and dual-luciferase reporter assays were used to confirm the binding between ITGB6 and latent TGFβ1. Transwell invasion, adherence and 3D tumor spheroid invasion assays were employed to test the effects of TGFβ1 on the invasion and adhesion of ovarian cancer spheroids. ELISA was performed to assess the release of latent TGFβ1 from ovarian cancer spheroids.

**Results:**

SMYD3 and ITGB6 activated the TGFβ1/Smad3 pathway and then induced the upregulation of Snail, Vimentin and N-cadherin and the downregulation of E-cadherin in 3D-cultured ovarian cancer spheroids. In this process, latent TGFβ1 could bind to ITGB6 and become activated to stimulate the Smad3 pathway. Moreover, SMYD3 and ITGB6 could facilitate the release of latent TGFβ1 from 3D-cultured ovarian cancer spheroids. Interestingly, TGFβ1 could promote the expression of SMYD3 and ITGB6 *via* feedback. This positive feedback loop could further amplify the biological effect and promote the invasion and adhesion of ovarian cancer spheroids.

**Conclusion:**

Our results demonstrated that the SMYD3/ITGB6/TGFβ1-Smad3 positive feedback loop could promote the invasion and adhesion of ovarian cancer spheroids by upregulating the expression of N-cadherin, Snail, and Vimentin and downregulating the expression of E-cadherin. Thus, our study unmasked the mechanism of SMYD3- and ITGB6-induced ovarian cancer metastasis and provides new ideas for targeted ovarian cancer treatment.

## Introduction

Ovarian cancer is the most lethal gynecological malignancy worldwide, and the lifetime risks of developing and dying from ovarian cancer are 0.72% and 0.45%, respectively (1, 2). Characteristically, over 70% of epithelial ovarian cancer (EOC) patients have intra-abdominal dissemination involving local invasion of pelvic and abdominal organs but rarely have vascular metastasis at diagnosis (3). This is mainly because of the unique characteristics of implantation metastasis in EOC, including detachment from the original lesion, formation of spheroids in ascites, adherence to peritoneal mesothelial cells, and anchoring to the stroma. In addition, biological features such as the adhesion and invasion potential of spheroid cells also account for EOC recurrence (4). Therefore, more research has cast light on the mechanism of EOC implantation metastasis in recent years. The phenotype and gene expression profile of EOC cells constantly change during the processes of shedding, suspension, re-adhesion and proliferation to adapt to the microenvironment.

SET and MYND domain-containing protein 3 (SMYD3) is a histone methyltransferase that acts as a gene transcriptional regulator, participating in the methylation of various histone and nonhistone targets (5). Aberrant SMYD3 expression is found during carcinogenesis in multiple cancers, such as prostate cancer, breast cancer and colorectal cancer, suggesting its essential roles in tumor initiation and progression (6).

In our previous studies, we found that compared to ovarian cancer cells grown in monolayers, ovarian cancer spheroids exhibited increased SMYD3 expression associated with higher levels of H3K4me3 (5). The SMYD3-H3K4me3-integrin pathway plays an important role in the pathogenesis of implantation metastasis in ovarian cancer. SMYD3-induced enhancement of spheroid invasion and adhesion is mediated by the downstream effectors ITGB6 and ITGAM (7). However, the potential mechanisms underlying SMYD3/integrin-mediated invasion and adhesion still need to be explored.

It is widely accepted that TGFβ plays a critical role in tumor progression and can mediate tumor immune escape, promote epithelial-mesenchymal transition (EMT) and help cells differentiate into more aggressive phenotypes for metastasis (8, 9). TGFβ1 is upregulated in ovarian cancer tissues and is associated with tumor progression, chemotherapy resistance and a poor prognosis (10). Nevertheless, more studies on the role of TGFβ1 in ovarian cancer metastasis are required.

TGFβ1 is secreted by cancer cells or fibroblasts in an inactive form, which consists of the 12.5-kDa carboxy-terminal region of TGFβ1 and a 25-kDa amino-terminal latency-associated peptide (LAP) joined by noncovalent bonding. This configuration prevents TGFβ1 from binding to its receptor (11). An acidic pH, reactive oxygen species, various kinases, thrombospondin-1 (THBS-1) and shear stress can activate latent TGFβ1 (12, 13). In a study on pulmonary fibrosis, ITGB6 was found to be the key activator of TGFβ1 in lung epithelial cells (14). According to reports and our research base, we speculated that SMYD3/ITGB6 promotes the invasion and adhesion of ovarian cancer spheroids by changing the configuration of TGFβ1-LAP and then activating TGFβ1.

In this report, we found that SMYD3 and ITGB6 could promote the release and activation of latent TGFβ1 and increase the phosphorylation of Smad3 activated by TGFβ1 in ovarian cancer spheroids. As downstream targets of the TGFβ1/SMAD3 pathway and essential participants in EMT promotion, N-cadherin, Vimentin, E-cadherin and Snail were found to be regulated by SMYD3 and ITGB6 and could contribute to enhanced invasion and adhesion in ovarian cancer spheroids. In addition, TGFβ1 could also upregulate the expression of SMYD3 and ITGB6 in return to form a positive feedback loop with the SMYD3/ITGB6/TGFβ1 pathway to enhance the metastasis of ovarian cancer spheroids.

## Materials and Methods

### Cell Lines and Culture Conditions

The human EOC cell line HEY was a gift from Dr. Robert Bast’s laboratory at the University of Texas Anderson Cancer Center, Houston, TX. The human EOC cell line A2780 were obtained from the Shanghai Key Laboratory of Female Reproduction Endocrine Related Diseases, Obstetrics and Gynecology Hospital, Fudan University. HEY and A2780 cells were cultured in complete RPMI 1640 medium at 37°C in a 5% CO_2_ environment. All complete media were supplemented with 10% fetal bovine serum (FBS), 100 U/ml penicillin and 100 µg/ml streptomycin.

For adherent culture (2D culture), cells were cultured in 10-cm common culture dishes (Corning, 430167) or common 6-well plates (Corning, 3516).

For suspended culture (3D culture), cells were cultured in 10-cm ultralow-attachment culture dishes (Corning, 3262) or ultralow-attachment 6-well plates (Corning, 3471) for 3 days.

### siRNA-Mediated Gene Silencing

A SMYD3-specific siRNA duplex (si-SMYD3, sequence: 5’-CCACAAGCGGGAAUGCAAA-3’) was synthesized by Genomeditech (Shanghai, China). NC-SMYD3 (5’-UUCUCCGAACGUGUCACGUdTdT-3’) was used as a negative control. An ITGB6-specific siRNA duplex (si-ITGB6, targeting the sequence 5’-GUCAAAGGAUGUCAAUUAATT-3’) was synthesized by GenePharma (Shanghai, China). NC-ITGB6 (5’-UUCUCCGAACGUGUCACGUTT-3’) was the corresponding negative control. An ITGAV-specific siRNA duplex (si-ITGAV, sequence: 5’-GAAUAUCGGUUGGAUUAUA-3’) was synthesized by Genomeditech (Shanghai, China). NC-ITGAV (5’-UUCUCCGAACGUGUCACGUdTdT-3’) was used as a negative control. For siRNA transfection, 1.5*10^5^ HEY cells/well or 3*10^5^ A2780 cells/well were seeded in a 6-well plate. The next day, we used Lipofectamine 3000 reagent (Invitrogen) for transient transfection according to the manufacturer’s protocol. Six to twelve hours later, the cells were trypsinized, counted and seeded in 2D and 3D culture systems for further experiments.

### Western Blotting

Antibodies against SMYD3 (Abcam, ab817149), ITGB6 (Abcam, ab187155), Smad3 (Abcam, ab40854), p-Smad3 (Abcam, ab52903), Snail (Cell Signaling Technology (CST), C15D3), N-cadherin (Abcam, ab76011), E-cadherin (CST, 3195), Vimentin (CST, 5741) and GAPDH (CST, 14C10) were used for western blotting. Cells were lysed in RIPA buffer containing 1:100 PMSF. A BCA protein assay kit (Solarbio, P0020) was used to measure the protein concentration. Equal amounts of protein were resolved by SDS-PAGE, transferred to PVDF membranes, and incubated with appropriate primary antibodies at the indicated concentration. Immune complexes were detected with HRP-conjugated secondary antibodies (Arigo Biolaboratories, ARG65351) and ECL chemiluminescence reagent (EpiZyme, SQ201). Each western blotting experiment was repeated at least two to three times. We quantified the western blot bands using ImageJ software.

### Cell Treatments Using an Inhibitor of and an Antibody Against TGFβ1

Cells were seeded in ultralow-attachment 6-well plates. For active TGFβ1 and SB431542 inhibitor treatment, 10 ng/ml recombinant human TGFβ1 (rhTGFβ1) protein (Abcam, ab50036) was added to the culture medium for 72 h, and the medium was replaced at 48 h. Then, 10 μM SB431542 (MedChemExpress, HY-10341) was added to the medium and incubated for 6 h (equal doses of PBS and DMSO were used as the negative controls for rhTGFβ1 and SB431542, respectively). The cells were collected for further western blotting and cell function assays. For the anti-ITGB6 inhibition assay, 10 μg/ml anti-ITGB6 antibody (Millipore, MAB2076Z) was added to the culture medium as a pretreatment to block ITGB6 for 12 h. Then, 10 ng/ml latent TGFβ1 (CST, 5154) was added to the culture medium for 72 h (equal doses of normal mouse IgG1 (Santa Cruz Biotechnology, sc-3877) and PBS were used as the negative controls for the anti-ITGB6 antibody and latent TGFβ1 experiments, respectively). The cells were collected for further western blotting and cell function assays.

### Transwell Invasion Assay

A Transwell system (24-well insert, pore size: 8 mm, Corning, 3422) was used to measure the invasive ability of 3D-cultured HEY cells. The inserts were coated with 50 μl of Matrigel (BD Bioscience Pharmingen, 356234) at a 1:8 dilution and incubated at 37°C overnight. The following day, cells were trypsinized, counted and resuspended in serum-free medium. A total of 1*10^4^ cells/200 μl of serum-free medium were added to the upper well of the chamber. In addition, 600 μl of complete medium was added to the lower well. After incubation for 16 h, the upper surface of the membrane was wiped with cotton swabs to remove any remaining cells, and the cells on the lower surface of the membrane were fixed with 4% paraformaldehyde and stained with 2% crystal violet. Five representative fields of each insert were imaged and counted using an Olympus light microscope at 100× magnification.

### Adhesion Assay

A 24-well plate was coated with 200 μl of Matrigel at a 1:50 dilution and air-dried in a biosafety cabinet for 6 h. Then, for blocking non-specific binding, 200 μl of serum-free medium containing 0.1% BSA (bovine serum albumin, Mpbio, 02FC007710) for 1 h. 3D-cultured cells were trypsinized, counted and resuspended in serum-free medium. A total of 3*10^4^ cells/200 μl of serum-free medium were added to each Matrigel-coated well and incubated in the incubator for 1-2 h. Nonadherent cells were removed by washing with PBS. Adherent cells were fixed with 4% paraformaldehyde and stained with 2% crystal violet. Five representative fields of each insert were imaged and counted using an Olympus light microscope at 40× magnification.

### 3D Tumor Spheroid Invasion Assay

This assay was performed as previously described (15). HEY cells (2×10^4^ cells/ml) were plated in ultralow-attachment 96-well round-bottomed plates in 200 μl of medium/well. rhTGFβ1 protein (10 ng/ml) was added to treat the cells in the TGFβ1+DMSO and TGFβ1+ SB431542 groups. The same volume of PBS was added to treat cells in the PBS+DMSO and PBS+SB431542 groups. After 3 days of incubation, 100 μl of culture medium was gently removed from each well, and 100 μl of Matrigel matrix was gently dispensed into each bottom well. The plate was transferred into a 37°C incubator, and the Matrigel matrix, which contained 20 ng/ml rhTGFβ1 or 20 μM SB431542 according to the experimental design, was allowed to solidify. One hour later, 100 μl of serum-free growth medium containing 10 ng/ml rhTGFβ1 or 10 μM SB431542 was gently added to each well. An image was recorded for each tumor spheroid at 0, 24, and 48 h to dynamically observe the 3D tumor spheroid invasion ability. Representative fields of spheroids were randomly counted using an Olympus light microscope at 100× magnification. The diameters of the spheroids were measured by ImageJ software.

### Dual-Luciferase Reporter Assay

A fragment of the PAI-1 promoter (-799~71 bp) was amplified and cloned into the pGL3 vector, which contains firefly luciferase (pGL3-PAI-1). Luciferase activity was measured using a dual-luciferase reporter assay system at 24h posttransfection according to the manufacturer’s instructions (Promega, E1910). Normalized data were calculated as the ratio of firefly/renilla luciferase activities.

### ELISA

The TGFβ1 concentration in the supernatant was measured with a Quantikine ELISA Human TGFβ1 kit (R&D Systems). A2780 cells were first subjected to siRNA-mediated gene silencing treatment for 48 h. A total of 6×10^5^ cells were suspended in 1.5 ml of culture medium, added to a single well of a six‐well ultralow-attachment plate, and then cultured for 72 h. The culture medium was not changed during the culture period. Then, the culture medium was collected in a 1.5 ml conical tube and centrifuged at 3,000 rpm for 15 minutes. The concentration of TGFβ1 in the culture medium was determined using a human TGFβ1 Quantikine ELISA kit (R&D Systems). HCl and NaOH/HEPES were used to convert latent TGFβ1 into the activated form.

### Immunofluorescence Assay

Hey and A2780 spheroids were embedded with Matrigel matrix in confocal dishes. The cells were fixed with 4% formaldehyde for 24 h, and blocked with 4% goat serum for 1 h at room temperature. The cells were then incubated with antibodies against E-cadherin (CST, 3195, 1:200), N-cadherin (CST, 13116, 1:200), at 4°C overnight. The dishes and slides were washed and incubated with Alexa Fluor 594 AffiniPure Donkey Anti- Rabbit IgG (H+L) (Yeasen, 34212ES60, 1:100) for 1 hr at room temperature. For F-actin, the cells were treated with 0.5% Triton (Absin, abs47048168) for 5 min and then, were incubated with Phalloidin (Yeasen, 40734ES75, 1:200) for 30 min at room temperature. Finally, the dishes were stained with DAPI (Beyotime, C1002, 1:1000) for 5 min. The fluorescently labeled cells (dishes and slides) were examined under a confocal laser scanning microscope (Zeiss LSM 880 Confocal Microscope, Germany) at room temperature.

### Statistical Analysis

Statistical analysis and graphing were conducted using GraphPad Prism 8. ImageJ was used for protein quantification. Statistically significant differences were determined by Student`s t-test, and p values <0.05 were considered significant.

## Results

### Key EMT Factors Are Promoted Along With SMYD3 and ITGB6 Upregulation During Ovarian Cancer Cell Spheroid Formation

**Figure 1:**
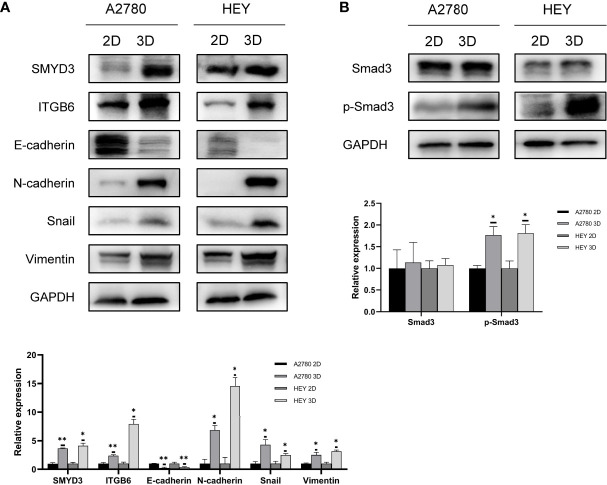
EMT along with SMYD3 and ITGB6 upregulation is promoted during ovarian cancer cell spheroid formation. **(A)** The expression levels of SMYD3, ITGB6, N-cadherin, Snail, Vimentin and E-cadherin in 2D- and 3D-cultured HEY and A2780 cells were evaluated by western blot analysis. **(B)** The phosphorylation level of Smad3 in 2D- and 3D-cultured HEY and A2780 cells was evaluated by western blot analysis. In the histogram of the western blot quantification, “*” refers to p < 0.05 and “**” refers to p < 0.01.

According to our previous study, SMYD3 is overexpressed in ovarian cancer ascites spheroids compared with primary ovarian cancer tissues. Using a 3D culture model to mimic the suspended growth conditions in ascites, we found that SMYD3 could enhance the adhesion and invasion of ovarian cancer spheroids and promote metastasis of ovarian cancer *in vivo* by upregulating the expression of ITGB6 and ITGAM (7). Compared with 2D-cultured HEY and A2780 cells, the corresponding 3D-cultured cells showed higher expression of SMYD3 and ITGB6, which indicated that the ovarian cancer spheroids had a more invasive phenotype. In addition, we found that the expression of N-cadherin, Snail, Vimentin and E-cadherin, which are essential molecules in the EMT process, was also increased in 3D-cultured HEY and A2780 cells (**Figure 1A**). Tumor cells can be more invasive during the EMT process. With this in mind, what is the mechanism of the upregulation of N-cadherin, Snail, and Vimentin and downregulation of E-cadherin during 2D-cultured ovarian cancer cell transformation into 3D spheroids? Do these EMT-related genes contribute to SMYD3/ITGB6-mediated spheroid metastasis? Both these questions need to be answered.

Smad3, as a well-known downstream signal of TGFβ1, can be activated by phosphorylated ALK5 after binding of TGFβ1 to its receptor (16). Since the TGFβ1/Smad3 signal transduction pathway is involved in inducing EMT in ovarian cancer (17), we aimed to identify whether the TGFβ1/Smad3 pathway is more activated in 3D-cultured ovarian cancer spheroids than in 2D-cultured ovarian cancer cells. As shown in **Figure 1B**, higher phosphorylation levels of Smad3 were found in 3D-cultured HEY and A2780 cells than in 2D-cultured cells, which indicated that TGFβ1/Smad3 might play a role in promoting the EMT process in ovarian cancer spheroids.

### Activation of the TGFβ1/Smad3 Pathway Is Conducive to the Regulation of EMT-Related Genes in 3D-Cultured Ovarian Cancer Spheroids

**Figure 2:**
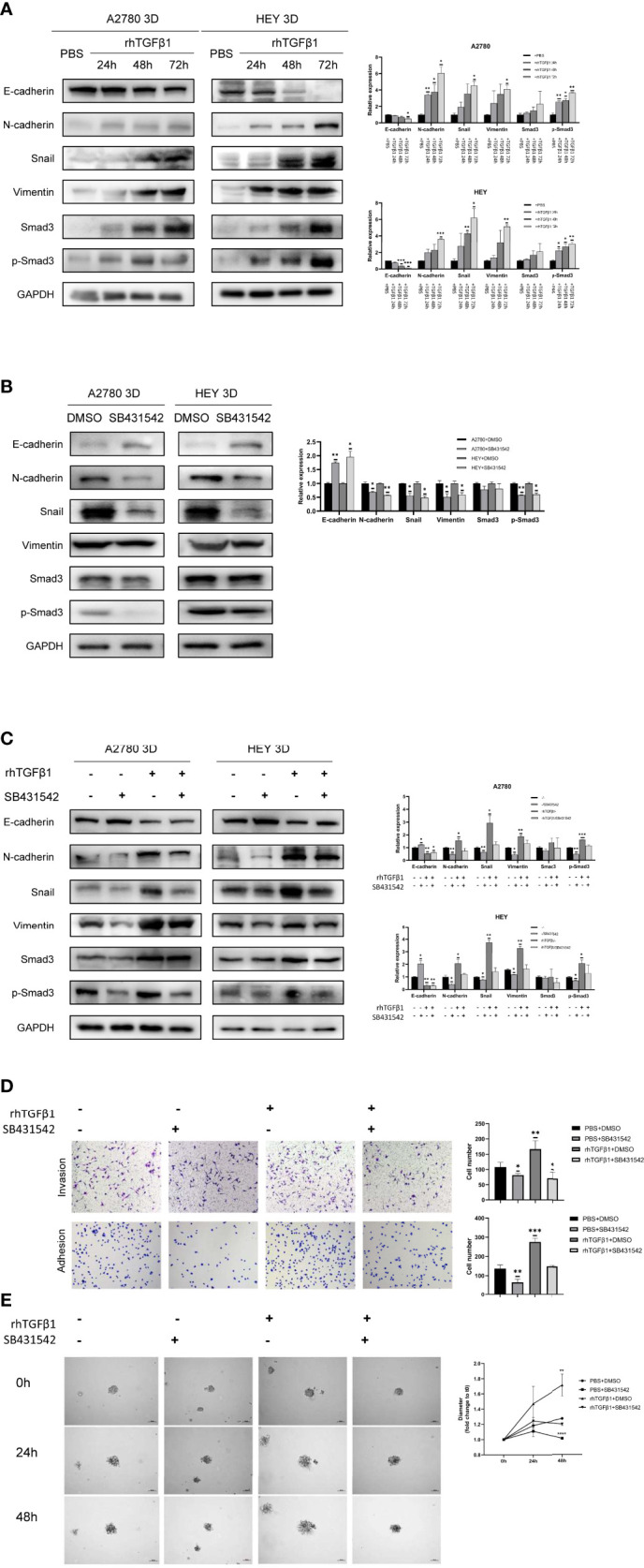
Activation of the TGFβ1/Smad3 pathway is conducive to the upregulation of EMT-related genes in 3D-cultured ovarian cancer spheroids. **(A)** Changes in the expression of N-cadherin, Snail, Vimentin and E-cadherin and the phosphorylation level of Smad3 in 3D-cultured HEY and A2780 cells after treatment with 10 ng/ml rhTGFβ1 protein for 24, 48 and 72 h **(B)** Changes in the expression of N-cadherin, Snail, Vimentin and E-cadherin and the phosphorylation level of Smad3 in 3D-cultured HEY and A2780 cells after treatment with 10 μM SB431542 for 6h. **(C)** Changes in the expression of N-cadherin, Snail, Vimentin and E-cadherin and the phosphorylation level of Smad3 in 3D-cultured HEY and A2780 cells after treatment with or without rhTGFβ1 and SB431542. RhTGFβ1 protein (10 ng/ml) was added to the culture medium for 72 h Then, 10 μM SB431542 was added to the medium and incubated for 6 h (equal doses of PBS and DMSO were used as the negative controls for rhTGFβ1 and SB431542, respectively). **(D)** Transwell invasion and adhesion assays revealed changes in the invasion and adhesion of 3D-cultured HEY cells after treatment with or without rhTGFβ1 and SB431542. **(E)** 3D tumor spheroid invasion assays revealed changes in the invasion and adhesion of 3D-cultured HEY cells after treatment with or without rhTGFβ1 and SB431542. RhTGFβ1 protein (10 ng/ml) was added to treat cells in the rhTGFβ1+DMSO and rhTGFβ1+ SB431542 groups. The same volume of PBS was added to treat cells in the PBS+DMSO and PBS+SB431542 groups. After 3 days of incubation, 100 μl of culture medium was gently removed from each well, and 100 μl of Matrigel matrix was gently dispensed into each bottom well. Matrigel matrix containing 20 ng/ml rhTGFβ1 was used in the rhTGFβ1+DMSO and rhTGFβ1+ SB431542 groups, and 20 μM SB431542 was used in the rhTGFβ1+ SB431542 and PBS+SB431542 groups. One hour later, 100 μl of serum-free growth medium containing 10 ng/ml rhTGFβ1 was gently added into each well of the rhTGFβ1+DMSO and rhTGFβ1+ SB431542 groups. SB431542 (10 μM) was gently added to the rhTGFβ1+SB431542 and PBS+SB431542 groups. “*” refers to p < 0.05, “**” refers to p < 0.01, “***” refers to p < 0.001, and “****” refers to p <0 .0001.

A review of the previous literature about the induction of EMT by TGFβ1 showed that the epithelial markers E-cadherin was repressed while the mesenchymal markers Vimentin and N-cadherin were induced during the induction of EMT by TGFβ1 (18). Since phosphorylated Smad3, N-cadherin, Snail and Vimentin were all increased in 3D-cultured ovarian cancer spheroids and E-cadherin was decreased in 3D-cultured ovarian cancer spheroids, we aimed to determine whether N-cadherin, Snail, Vimentin and E-cadherin are target molecules of the activated Smad3 pathway in 3D-cultured ovarian cancer spheroids. First, we used rhTGFβ1 to treat 3D-cultured HEY and A2780 cells for 24, 48 and 72 hours. Over time, the amount of phosphorylated Smad3 increased under the condition of constant expression of total Smad3, which demonstrated that TGFβ1 could stimulate the Smad3 pathway. In addition, the expression of N-cadherin, Snail and Vimentin was also increased and the expression of E-cadherin was decreased after treatment with rhTGFβ1 (**Figure 2A**). Subsequently, SB431542, an inhibitor of ALK5, was added to the supernatant of 3D-cultured HEY and A2780 cells and incubated for 6 hours. As the phosphorylation of Smad3 was inhibited by SB431542, the expression of N-cadherin, Snail and Vimentin also declined, and the expression of E-cadherin was enhanced (**Figure 2B**). Furthermore, we found that the regulation of EMT-related genes induced by rhTGFβ1 could be restrained by SB431542 (**Figure 2C**). These findings showed that TGFβ1 could stimulate the Smad3 pathway and contribute to the increases in the expression of Snail and N-cadherin. Finally, we used Transwell invasion, adherence and 3D tumor spheroid invasion assays to validate the functional effects of rhTGFβ1 and SB431542 on the EMT process. RhTGFβ1 enhanced the invasion and adhesion of 3D-cultured HEY cells, and these effects were inhibited when SB431542 was added simultaneously (**Figures 2D, E**).

### SMYD3 and ITGB6 Can Activate the TGFβ1/Smad3 Pathway and Regulate the Expression of EMT-Related Genes in 3D-Cultured Ovarian Cancer Spheroids

**Figure 3:**
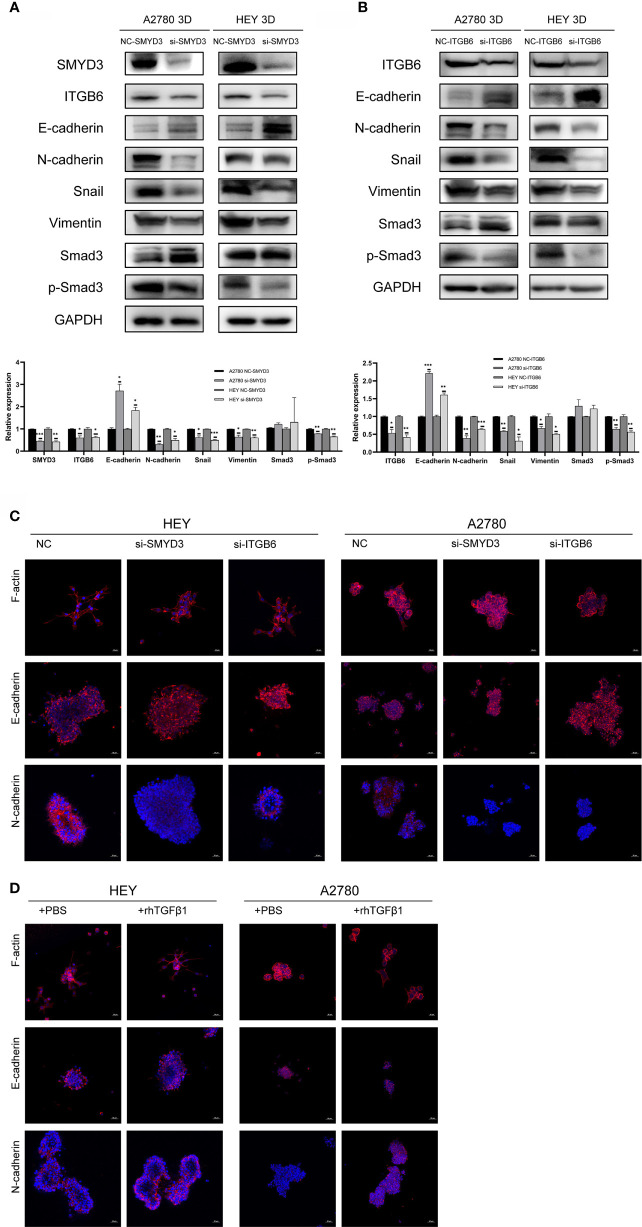
SMYD3 and ITGB6 can activate the TGFβ1/Smad3 pathway and upregulate the expression of EMT-related genes in 3D-cultured ovarian cancer spheroids. **(A)** The changes in the phosphorylation level of Smad3 and the expression of SMYD3, ITGB6, N-cadherin, Snail, Vimentin and E-cadherin in 3D-cultured HEY and A2780 cells were evaluated by western blot analysis after SMYD3 silencing. **(B)** The changes in the phosphorylation level of Smad3 and the expression of ITGB6, N-cadherin, Snail, Vimentin and E-cadherin in 3D-cultured HEY and A2780 cells were evaluated by western blot analysis after ITGB6 silencing. **(C)** Immunofluorescence assay exhibited the expression changes of F-actin, E-cadherin and N-cadherin in both 3D-cultured HEY and A2780 cells after silencing SMYD3 and ITGB6, respectively. **(D)** Immunofluorescence assay exhibited the expression changes of F-actin, E-cadherin and N-cadherin in both 3D-cultured HEY and A2780 cells after rhTGFβ1 treatment. In the histogram of the western blot quantification, “*” refers to p < 0.05, “**” refers to p < 0.01, and “***” refers to p < 0.001.

To determine why SMYD3 and ITGB6 could make ovarian cancer spheroids more invasive, we downregulated the expression of SMYD3 and ITGB6 in 3D-cultured HEY and A2780 cells. As shown in **Figure 3A**, after SMYD3 silencing, the expression of its target gene ITGB6 was also decreased. In addition, the phosphorylation levels of Smad3 were consequently reduced, along with decreased expression of N-cadherin, Snail, and Vimentin and increased expression of E-cadherin. After ITGB6 silencing, the expression changes in p-Smad3, N-cadherin, Snail, Vimentin, E-cadherin were consistent with those observed with SMYD3 silencing (**Figure 3B**). In addition, using immunofluorescence assay, we also demonstrated that when we inhibited the expression of SMYD3 and ITGB6, the expression of E-cadherin significantly increased. And as the 3D-cultured ovarian cells were treated with rhTGFβ1, it significantly decreased. The expression of N-cadherin showed opposite tendency to the one of E-cadherin (**Figures 3C, D**).

Considering to the expression changes of EMT-related genes, we should also pay attention to the morphological changes of 3D-cultured ovarian cancer cells. The epithelial-like structure of 3D-cultured spheroid is more solid round. And the mesenchymal-like structure of 3D-cultured is grape-like or spindle like (19). The 3D-cultured HEY and A2780 spheroid with SMYD3 or ITGB6 silencing presented the robust cell-cell adherence and more solid structure compared with 3D-cultured HEY and A2780-NC spheroid (**Figure 3C**). The 3D-cultured HEY and A2780 spheroid presented the loose cell-cell adherence and spindle-like structure when it was exposed to rhTGFβ1 (**Figure 3D**). Therefore, SMYD3 and ITGB6 could stimulate the TGFβ1/Smad3 pathway and regulate the expression of N-cadherin, Snail, Vimentin, E-cadherin in ovarian cancer spheroids and promote the EMT process.

### Latent TGFβ1 Can Bind to ITGB6 and Release Active TGFβ1 to Stimulate the Smad3 Pathway

**Figure 4:**
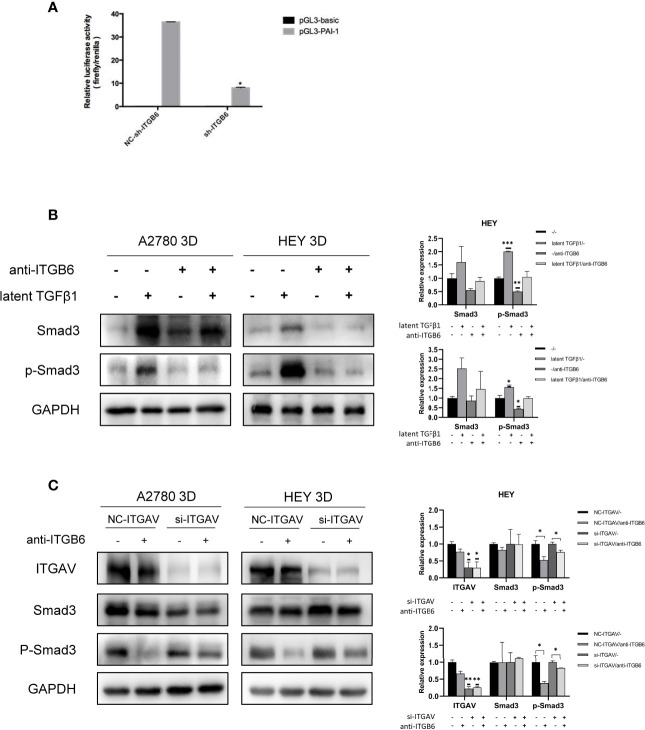
Latent TGFβ1 can bind to ITGB6 and release active TGFβ1 to stimulate the Smad3 pathway. **(A)** A dual-luciferase reporter assay showed changes in 3D-cultured HEY cells after ITGB6 silencing. **(B)** Changes in the phosphorylation level of Smad3 in 3D-cultured HEY and A2780 cells after treatment with or without latent TGFβ1 and anti-ITGB6 antibody. Anti-ITGB6 antibody (10 μg/ml) was added to the culture medium as a pretreatment to block ITGB6 for 12 h. Then, 10 ng/ml latent TGFβ1 was added to the culture medium for 72 h (equal doses of normal mouse IgG1 and PBS were used as the negative controls for the anti-ITGB6 antibody and latent TGFβ1, respectively). **(C)** Changes in the phosphorylation level of Smad3 in 3D-cultured HEY and A2780 cells after silencing of ITGAV. NC-ITGAV and si-ITGAV were transfected into HEY and A2780 cells. After 6 h, the cells were resuspended in ultralow-attachment plates for 3D culture. Then, 10 μg/ml anti-ITGB6 antibody was used to block ITGB6 for 12 h in 3D-cultured NC-ITGAV and si-ITGAV cells, and equal doses of normal mouse IgG1 were used as a negative control. Subsequently, 10 ng/ml latent TGFβ1 was added to the culture medium and incubated for 72 h in each group. In the histogram of the quantification, “*” refers to p < 0.05, “**” refers to p < 0.01, and “***” refers to p < 0.001.

How can SMYD3 and ITGB6 stimulate the TGFβ1/Smad3 pathway? TGFβ1 is produced as a latent precursor and functions in an active form. Therefore, the activation of latent TGFβ1 is a crucial regulatory event. ITGB6 was reported to be important in stringently localized activator of latent TGFβ1 at epithelial surfaces (20). Therefore, we performed a dual-luciferase reporter assay to identify whether ITGB6 plays a role in the activation of TGFβ1. Plasminogen activator inhibitor-1 (PAI-1) is a well-known target gene of TGFβ1 (21). When we downregulated the expression of ITGB6 in 3D-cultured HEY cells, the relative luciferase activity of PAI-1 was decreased accordingly (p<0.0001) (**Figure 4A**). These results suggested that ITGB6 could stimulate the TGFβ1 pathway. Then, we used latent TGFβ1 to treat 3D-cultured HEY and A2780 cells. As a result, the Smad3 pathway was activated. However, after adding a monoclonal antibody against ITGB6 to inhibit latent TGFβ1 binding to ITGB6, latent TGFβ1 failed to stimulate the Smad3 pathway (**Figure 4B**). Therefore, latent TGFβ1 could be transformed into the active form by binding to ITGB6 and then induce the activation of the Smad3 pathway.

As the binding partner of ITGB6, ITGAV has also been reported to be able to activate latent TGFβ1 in tumors (22, 23). It is necessary to investigate whether ITGAV could regulate the activation of latent TGFβ1 in 3D-cultured ovarian cancer spheroids and whether ITGAV is involved in the ITGB6-regulated activation of latent TGFβ1 in 3D-cultured ovarian cancer spheroids. First, we silenced the expression of ITGAV in 3D-cultured ovarian cancer spheroids using siRNA. After treatment with latent TGFβ1, the phosphorylation level of Smad3 did not decrease in ITGAV knockdown cells. Therefore, ITGAV might have no effect on the activation of latent TGFβ1 in 3D-cultured ovarian cancer spheroids. Then, we used 10 μg/ml anti-ITGB6 antibody to block ITGB6 for 12 h in 3D-cultured NC-ITGAV and si-ITGAV cells. After that, 10 ng/ml latent TGFβ1 was added to the culture medium for 72 h. We found that anti-ITGB6 antibody was more effective in inhibiting the phosphorylation level of Smad3 in 3D-cultured NC-ITGAV cells than in 3D-cultured si-ITGAV cells, implying that ITGAV might play a role in ITGB6-regulated activation of latent TGFβ1 in 3D-cultured ovarian cancer spheroids (**Figure 4C**).

### SMYD3 and ITGB6 Can Facilitate the Release of Latent TGFβ1 From 3D-Cultured Ovarian Cancer Spheroids

**Figure 5:**
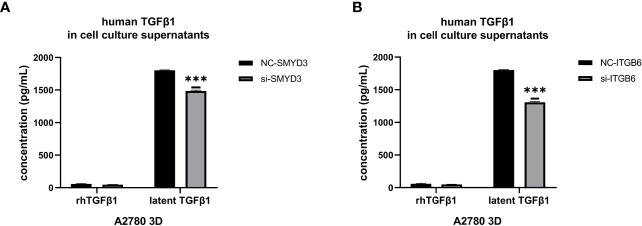
SMYD3 and ITGB6 can facilitate the release of latent TGFβ1 from 3D-cultured ovarian cancer spheroids. **(A, B)** ELISA was used to measure the concentrations of active TGFβ1 and latent TGFβ1 released from 3D-cultured A2780 cells after SMYD3 **(A)** or ITGB6 **(B)** silencing. In the histogram of the ELISA quantification, “***” refers to p < 0.001.

Since we found that SMYD3 and ITGB6 could upregulate the expression of Snail and N-cadherin *via* the activation of the TGFβ1/Smad3 pathway, whether SMYD3 and ITGB6 can enhance the release of latent TGFβ1 needed to be explored. We used ELISA to quantify the amounts of active and total TGFβ1, and the difference was considered the amount of latent TGFβ1. We found that the latent TGFβ1 level was decreased after downregulating SMYD3 or ITGB6 (both p<0.0001) (**Figures 5A, B**). Hence, SMYD3 and ITGB6 could not only activate latent TGFβ1 but also increase the release of latent TGFβ1 from 3D-cultured ovarian cancer spheroids.

### TGFβ1 Can Promote the Expression of SMYD3 and ITGB6 *via* Feedback

**Figure 6:**
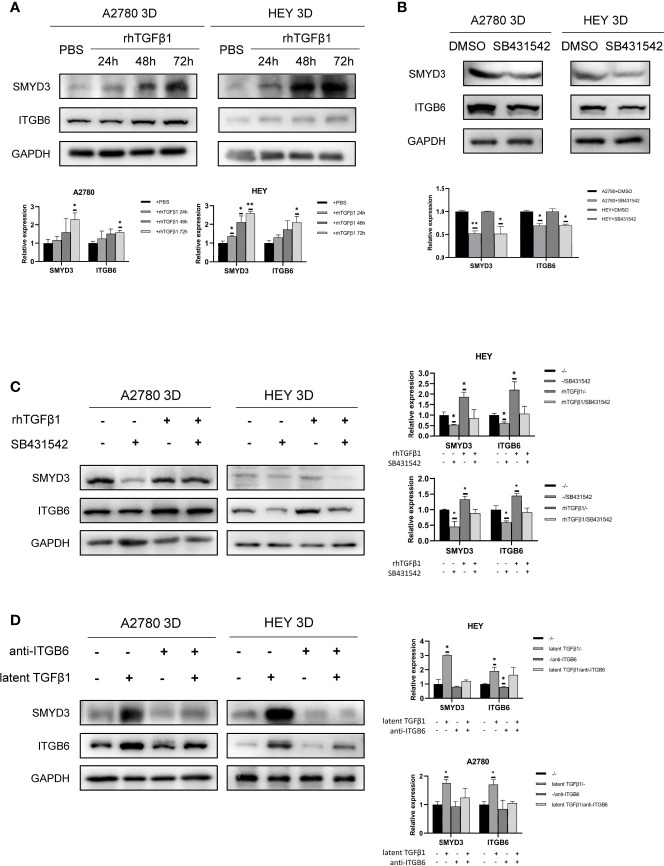
TGFβ1 can promote the expression of SMYD3 and ITGB6 *via* feedback. **(A)** Changes in the expression of SMYD3 and ITGB6 in 3D-cultured HEY and A2780 cells after treatment with 10 ng/ml recombinant human TGFβ1 protein for 24, 48 and 72 h. **(B)** Changes in the expression of SMYD3 and ITGB6 in 3D-cultured HEY and A2780 cells after treatment with 10 μM SB431542 for 24, 48 and 72 h. **(C)** Changes in the expression of SMYD3 and ITGB6 in 3D-cultured HEY and A2780 cells after treatment with or without rhTGFβ1 and SB431542. RhTGFβ1 protein (10 ng/ml) was added to the culture medium and incubated for 72 h. Then, 10 μM/ml SB431542 was added to the medium and incubated for 6 h (equal doses of PBS and DMSO were used as the negative controls for rhTGFβ1 and SB431542, respectively). **(D)** Changes in the protein expression of SMYD3 and ITGB6 in 3D-cultured HEY and A2780 cells after treatment with or without latent TGFβ1 and anti-ITGB6. In the histogram of the western blot quantification, “*” refers to p < 0.05 and “**” refers to p < 0.01.

We verified that ITGB6 could activate latent TGFβ1. Subsequently, we found that TGFβ1 could also promote the expression of SMYD3 and ITGB6 in 3D-cultured HEY and A2780 cells (**Figure 6A**). SB431542 reduced the expression of SMYD3 and ITGB6 (**Figure 6B**). The upregulation of SMYD3 and ITGB6 due to the activation of TGFβ1 could be inhibited by SB431542 (**Figure 6C**). These findings demonstrated that TGFβ1 promotes the expression of SMYD3 and ITGB6 *via* the Smad3 pathway. As shown in **Figure 6D**, latent TGFβ1 facilitated the expression of SMYD3 and ITGB6. After the binding of latent TGFβ1 to ITGB6 was blocked, the expression of SMYD3 and ITGB6 also declined. Thus, the SMYD3/ITGB6/TGFβ1 positive feedback loop drove the upregulation of Snail and N-cadherin, which promoted invasion, adhesion and EMT progression during ovarian cancer metastasis.

## Discussion

Ovarian cancer is the leading cause of death among gynecological malignancies. Worldwide, the incidence rate of ovarian cancer is seventh among those of female malignant cancers, while the mortality rate is eighth, and the 5-year survival rate is below 45% (24). The main metastatic mode of ovarian cancer is the transcoelomic pathway. Briefly, cells disseminate from the primary epithelial ovarian tumor and float as spheroids in the ascites. Metastatic cells then attach to peritoneal organs and form macrometastatic colonies (25). Both genetic changes and epigenetic changes contribute to the initiation and transcoelomic process of ovarian cancer. This disease is characterized by “genomic chaos” caused by high chromosomal instability due to a massive number of copy number abnormalities and chromosomal alterations (26, 27). Therefore, epigenetic enzymes may play a great role in regulating the transcoelomic spread of ovarian cancer.

According to the previous work of our team, SMYD3, a histone methylation transferase, was found to be expressed at higher levels in ascites-derived spheroids than in primary ovarian tumor cells from EOC patients. In ovarian cancer spheroids, SMYD3 was found bind to H3K4me3 at the ITGB6 and ITGAM promoter regions to upregulate the expression of ITGB6 and ITGAM, which was verified to enhance the invasion and adhesion of ovarian cancer spheroids (7). ITGB6 is an essential member of the integrin family. The integrin family is documented to be involved in various cancer processes (such as tumor initiation, metastasis and drug resistance), and ITGB6 is also reported to be associated with the progression and metastasis of oral squamous cell carcinoma, bladder cancer and colorectal carcinoma (28–31). Previously, we found that ITGB6 could promote the invasion and adhesion of ovarian cancer spheroids. However, the underlying molecular mechanisms should be further explored.

EMT is one of the phenotypic plasticity processes relevant to metastasis. During EMT, epithelial cells lose polarity and develop a stromal phenotype, increasing invasiveness. Cells with features of EMT are able to migrate, invade and undergo metastatic dissemination. TGFβ is a common inducer of the EMT process (32). TGFβ signaling promotes EMT by inducing the expression of several pleiotropic transcription factors, also known as “master regulators” of EMT (such as Snail, N-cadherin, E-cadherin and Vimentin). Proteins of the Smad family are the major effectors that control the transduction of intracellular signaling initiated by the TGFβ superfamily of cytokines. SMAD-dependent signaling can be activated by an active TGFβ1 ligand initially binding to TGFβRII, followed by recruitment of ALK5 (TGFβRI) to the plasma membrane (33). Here, we observed that in ovarian cancer spheroids, the Smad3 pathway was activated by TGFβ1 and upregulated the expression of Snail and N-cadherin, which are crucial genes in the EMT process.

The three isoforms of TGFβ (TGFβ1, TGFβ-2 and TGFβ-3) are always produced as inactive cytokines that cannot bind to their receptor and are not functional unless they are activated. An integrin-mediated mechanism makes an essential contribution to TGFβ activation *in vivo*. TGFβ activation has been demonstrated to be caused by the RGD tripeptide motif in the LAP region of latent TGFβ binding to specific integrin receptors (20). Khalid Puthawala et al. reported that integrin alpha(v)beta6-mediated TGFβ activation is required for radiation-induced lung fibrosis (34). Laura L Koth et al. found that integrin alpha(v)beta6-mediated TGFβ activation regulates the homeostasis of phospholipids and collectins in the lungs (35). To date, few articles have stated the role of ITGB6-mediated TGFβ activation in tumor progression. In our study, we found that downregulated expression of ITGB6 could reduce the activation level of the Smad3 pathway when ovarian cancer spheroids were treated with rhTGFβ1. When ovarian cancer spheroids were treated with latent TGFβ1, the activation of the Smad3 pathway could also be inhibited if ITGB6 was blocked by a specific antibody. After the expression of SMYD3 or ITGB6 was altered, the expression of N-cadherin, Snail, Vimentin and E-cadherin and the morphological structure of the spheroids were consequently changed. Hence, we determined that ITGB6-mediated TGFβ activation is involved in regulating the expression of N-cadherin, Snail, Vimentin and E-cadherin and promoting EMT progression in ovarian cancer spheroids. In addition, as the binding partner of ITGB6, ITGAV, had no independent effect on activating latent TGFβ1 in 3D-cultured ovarian cancer spheroids. However, ITGAV might be play a role in the ITGB6-regulated activation of latent TGFβ1 in 3D-cultured ovarian cancer spheroids. Therefore, in future, more studies should be done to explore the role of ITGAV in TGFβ1/SMAD signaling activation. Interestingly, attenuating the expression of SMYD3 and ITGB6 also decreased the release of latent TGFβ1 from ovarian cancer spheroids, which further demonstrated the regulatory roles of SMYD3 and ITGB6 in TGFβ1 pathway activation.

**Figure 7:**
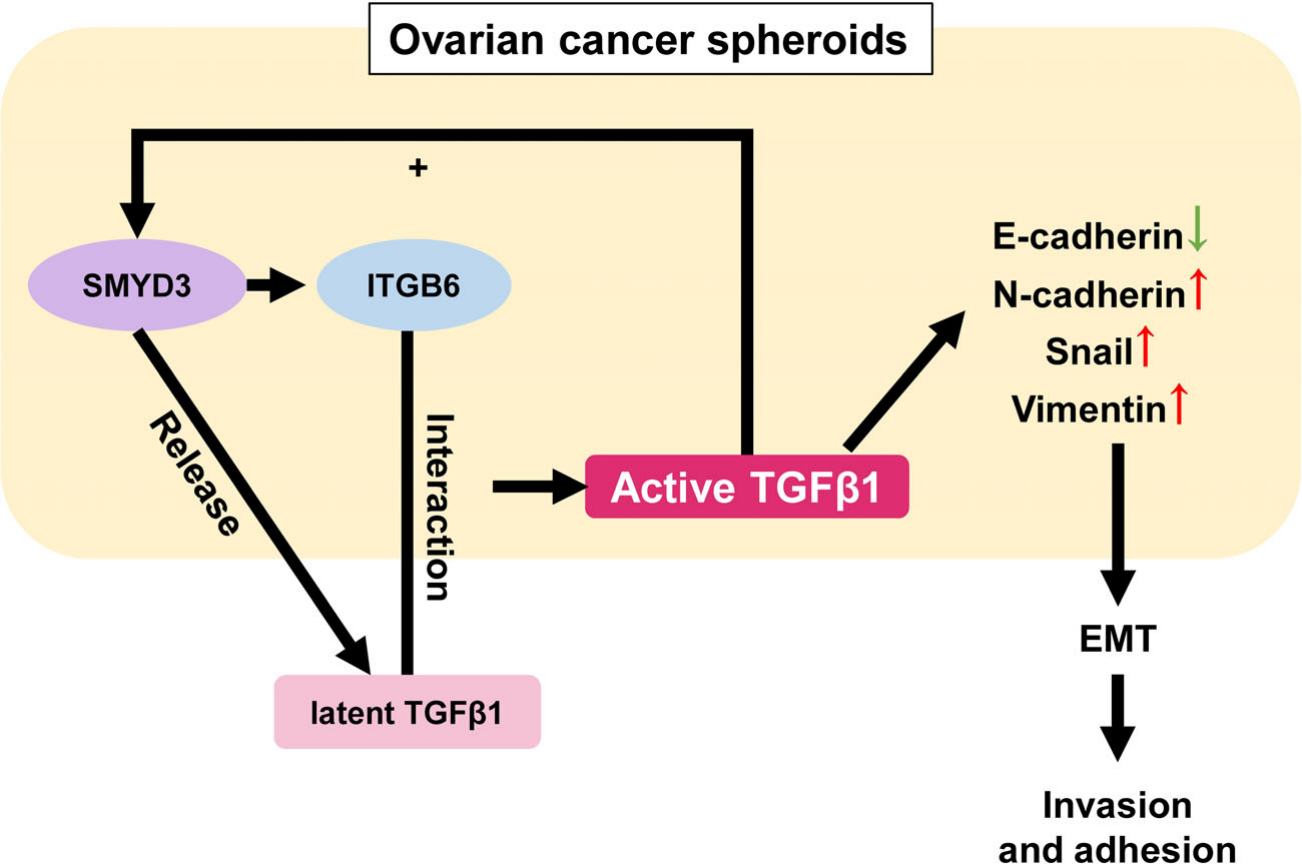
Schematic model of the role of the SMYD3/ITGB6/TGFβ1 positive feedback loop in promoting the invasion and adhesion of ovarian cancer spheroids. In ovarian cancer spheroids, SMYD3 can increase the expression of ITGB6. When latent TGFβ1 binds to ITGB6 on the cell surface, TGFβ1 is activated and upregulates the expression of N-cadherin, Snail, Vimentin as well as downregulates E-cadherin to enhance the invasion and adhesion of ovarian cancer spheroids by stimulating the Smad3 pathway. Ovarian cancer spheroids can release more latent TGFβ1 at the same time. In addition, TGFβ1 can also facilitate the expression of SMYD3 and ITGB6 in a feedback manner.

Surprisingly, we also found that the expression of SMYD3 and ITGB6 could be upregulated after TGFβ1 treatment, which formed a positive feedback loop among SMYD3/ITGB6/TGFβ1 to enhance the invasion and adhesion of ovarian cancer spheroids by regulating the expression of E-cadherin, N-cadherin, Snail and Vimentin (**Figure 7**). Several previous studies also showed that TGFβ could induce the expression of SMYD3 and ITGB6. Denise et al. stated that SMYD3 was specifically regulated by TGFβ in iTreg cells (36). Mingyan Xu et al. reported that TGFβ1 could induce ITGB6 transcription *via* JunB- and CBP-mediated histone hyperacetylation in oral squamous cell carcinoma (37). Chao Jing et al. reported that miR-17/20a could reduce the expression of ITGB6 by attenuating the activation of TGFβ and phosphorylation of SMAD2/3 in esophageal squamous cell carcinoma (38).

In conclusion, we detailed one essential pathway that contributed to the SMYD3- and ITGB6-mediated enhancement of invasion and adherence in ovarian cancer spheroids. Our study also emphasized the significance of choosing SMYD3 and ITGB6 as potential targets for the treatment of ovarian cancer transcoelomic metastasis.

## Data Availability Statement

The raw data supporting the conclusions of this article will be made available by the authors, without undue reservation.

## Ethics Statement

All experiments were approved by the Ethics Committee of Ruijin Hospital, Shanghai Jiao Tong University, School of Medicine. The informed consents were obtained from all patients included in the study.

## Author Contributions

All authors contributed to the article and approved the submitted version. YJ and TZ contributed in writing the manuscript, conducting the experiments, and doing the data analysis. YS helped to conduct part of the experiments. WF and TL designed this study and were the corresponding authors of this manuscript.

## Funding

This work was supported by the National Natural Science Foundation of China (grant number: 8217100345), Ruijin Youth NSFC Cultivation Fund (Grant No. 2019QNPY02014) and Guangci Distinguished Young Scholars Training Program of Shanghai Jiaotong University School of Medicine affiliated Ruijin Hospital (GCQN-2019-B12).

## Conflict of Interest

The authors declare that the research was conducted in the absence of any commercial or financial relationships that could be construed as a potential conflict of interest.

## Publisher’s Note

All claims expressed in this article are solely those of the authors and do not necessarily represent those of their affiliated organizations, or those of the publisher, the editors and the reviewers. Any product that may be evaluated in this article, or claim that may be made by its manufacturer, is not guaranteed or endorsed by the publisher.
